# Supplementary material for: A secreted antibacterial neuropeptide shapes the microbiome of Hydra
Source: Nat Commun. 2017 Sep 26;8:698. doi: 10.1038/s41467-017-00625-1 (PMC5614986; doi:10.1038/s41467-017-00625-1)
Supplement: Supplementary file 1 — Supplementary Information [file 41467_2017_625_MOESM1_ESM.pdf]

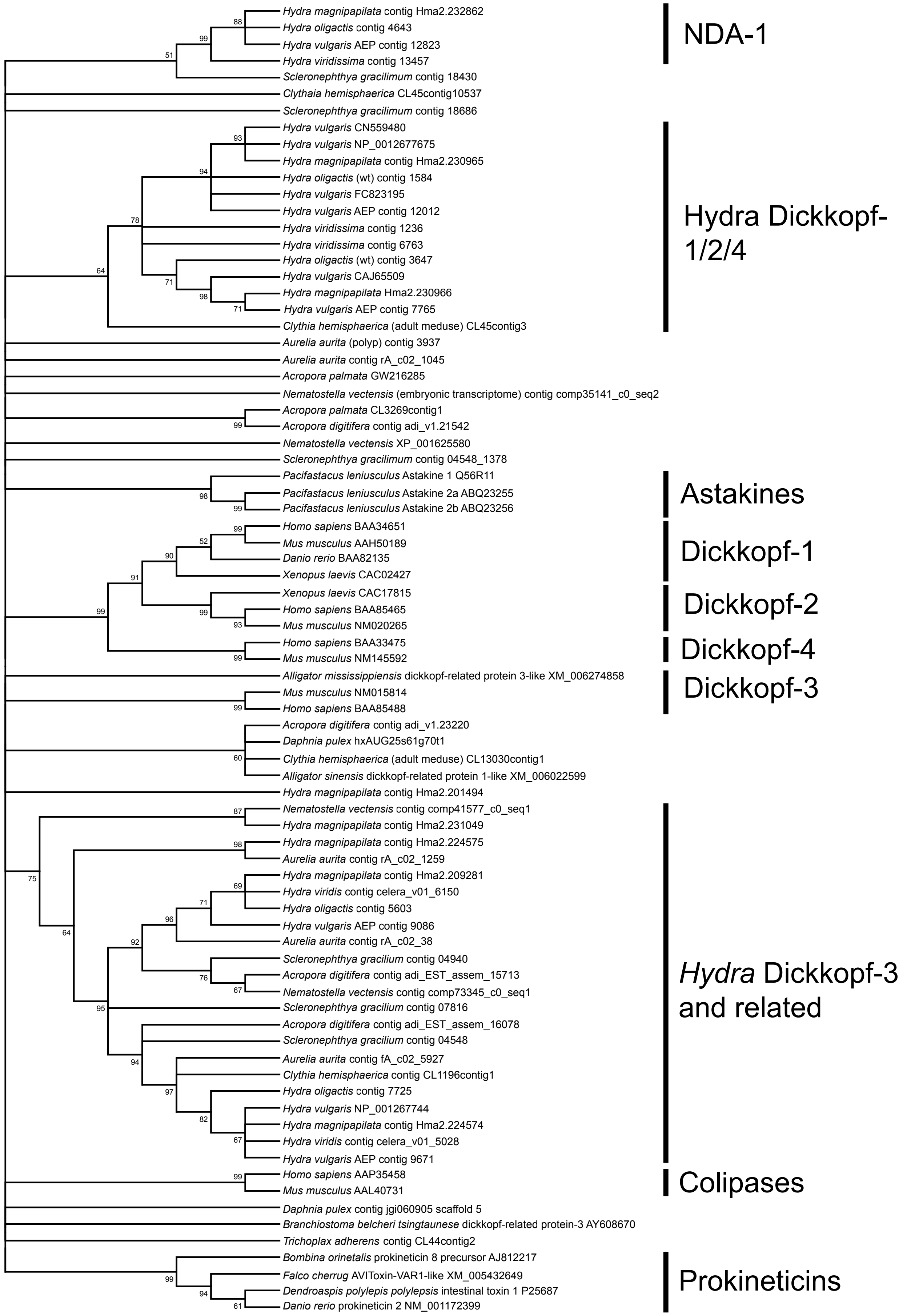

Supplementary Figure 1. Neighbour-joining phylogenetic tree of prokineticins, astakines, Dickkopf and colipase gene families showing absence of congruence between NDA-1 (at the top) and the other peptides.

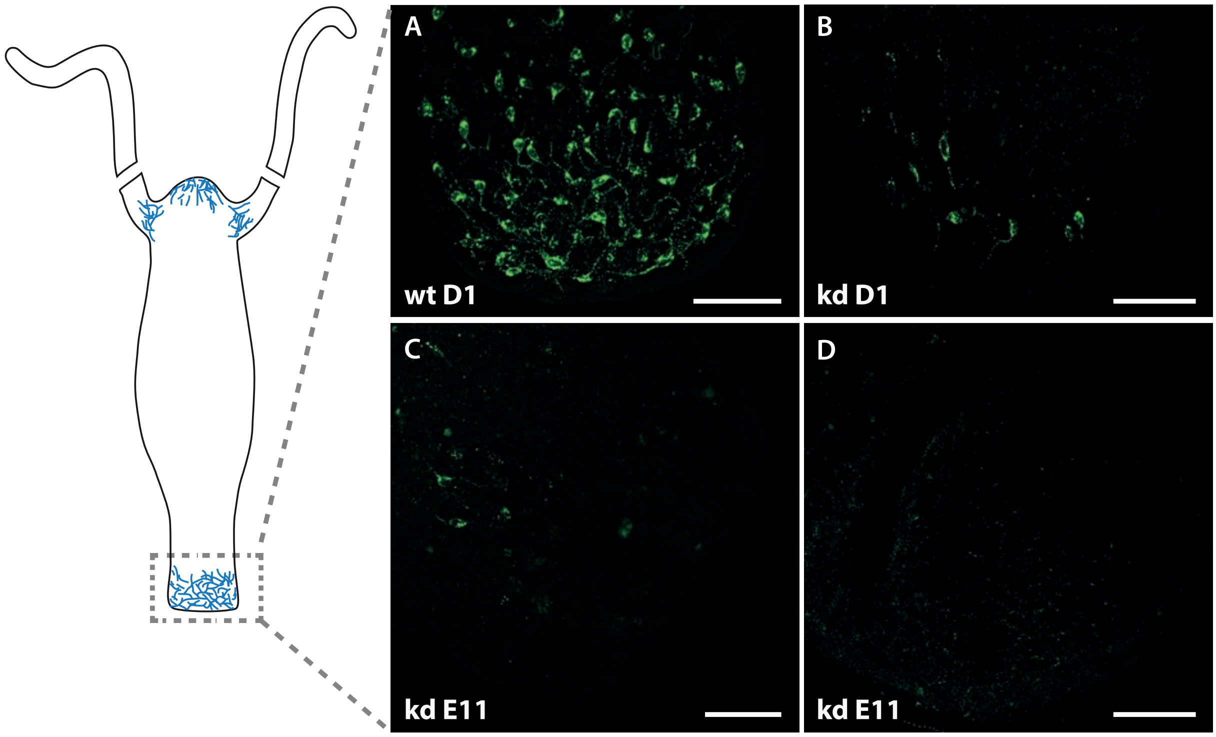

Supplementary Figure 2. Confocal laser scanning micrographs of the foot region of Hydra, stained for NDA-1 peptide by means of polyclonal antiserum. Wildtype (panel A) is compared to the transgenic knockdown lines D1 (B) and E11 (C, D), which have lower NDA-1 expression levels. Scale bars: 50 µm.

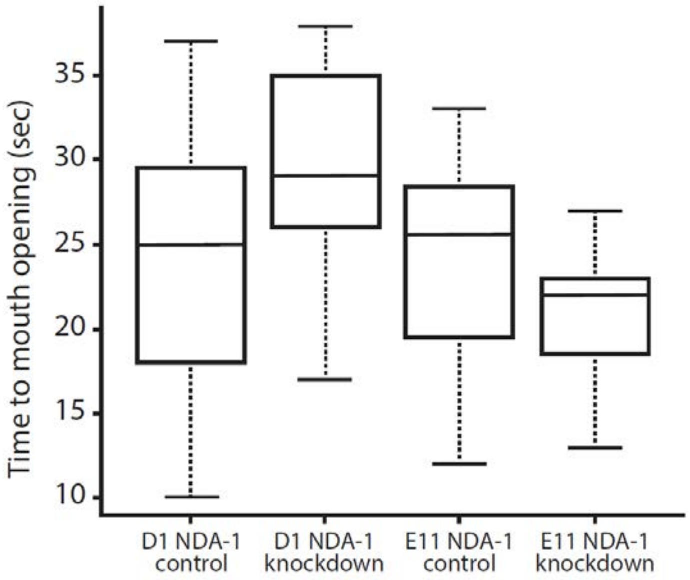

Supplementary Figure 3. Glutathione (GSH) response of NDA-1 knockdown and control polyps. The feeding response of *Hydra* can be evoked chemically using micromolar concentrations of γ-glutamil-cysteinyl-glycine (reduced glutathione, GSH), which forces the polyps to open their mouth as they do when capturing prey[1]. This GSH-induced response, like feeding behaviour, is neuron-dependent, since nerve-free animals do not open their mouth[2]. The figure shows the GSH response in 100% of NDA-1 knockdown animals after exposure to 10μM GSH. There was no statistical significant difference in time to mouth opening between NDA-1 knockdown and control animals (t-test: D1: $t = -2$, $df = 20$, $p = 0.1$; NDA-1 control $n = 12$, NDA-1 knockdown $n = 13$; E11: $t = 1$, $df = 20$, $p = 0.2$; NDA-1 control $n = 12$, NDA-1 knockdown $n = 12$). Boxes in boxplots represent the interquartile range, the line inside the box corresponds to the median, and the wiskers display the span of the data (maximun and minimum values)

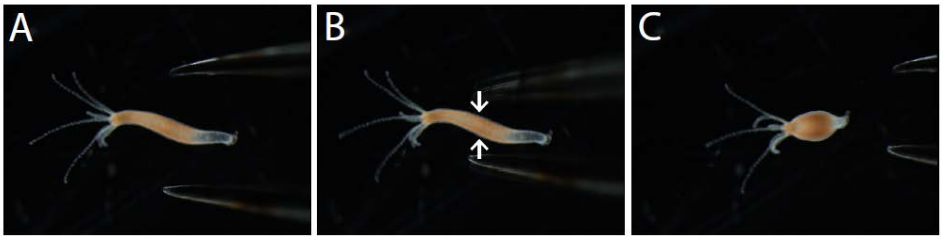

D

	D1 NDA-1 control	D1 NDA-1 knockdown	E11 NDA-1 control	E11 NDA-1 knockdown
Number of animals contracting	20 / 20	14 / 14	16 / 16	19 / 19

Supplementary Figure 4. Contractile behavior induced by mechanical stimulation.
When *Hydra* polyps are pinched with a pair of forceps, the animals response with rapid contraction of the body column[2]. Here this response to mechanical stimulation is shown. Panel A: no stimulation. Panel B: pincer stimulation as indicated by arrows. Panel C: contraction of body column. Panel D: Response of control and NDA-1 knockdown animals. There was no difference in contractile behaviour.

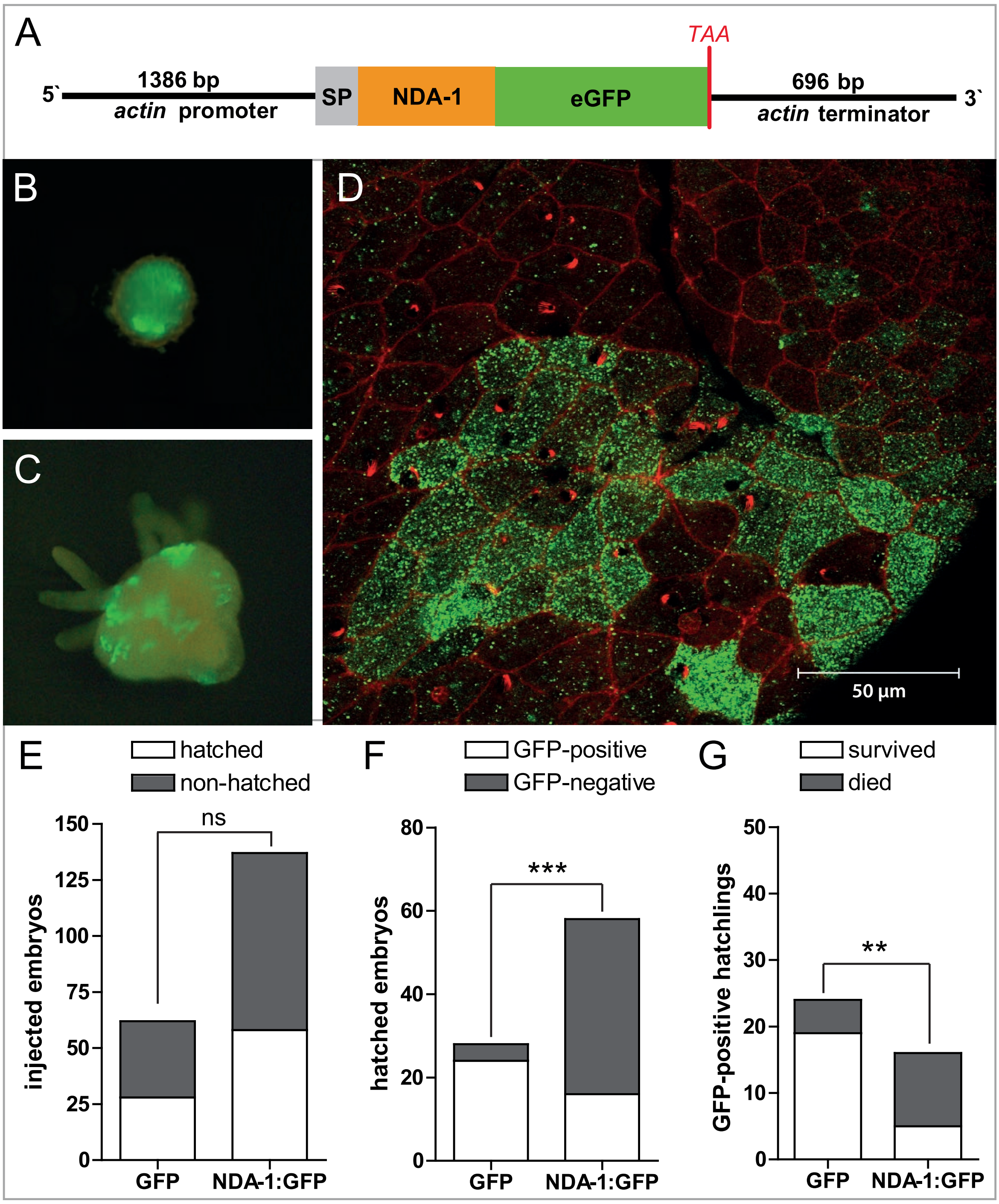

Supplementary Figure 5. Ectopic overexpression of NDA-1:eGFP.
(A) Construct used for generation of transgenic hydra overexpressing NDA-1:eGFP. **(B, C)** *In vivo* images of a transgenic *Hydra vulgaris* (AEP) embryo **(B)** and hatchling **(C)** with mosaic expression of NDA-1:eGFP. **(D)** Immunostaining detects the NDA-1:eGFP fusion protein in dense vesicles of ectodermal epithelial cells. Green, eGFP; red, rhodamin-phalloidin for F-actin. **(E)** Embryos injected with the NDA-1:eGFP construct showed no difference in hatching rate compared to embryos injected with a control construct based on the same vector lacking the NDA-1 sequence (eGFP: n=62; NDA-1:eGFP: n=137; p=0.7583, Fisher's exact test). **(F)** NDA-1:eGFP injection resulted in significantly less GFP-positive hatchlings compared to the control construct (eGFP: n=28; NDA-1:eGFP: n= 58; p<0.0001, Fisher's exact test). **(G)** Hatchlings expressing NDA-1:eGFP showed significantly reduced survival compared to hatchlings expressing eGFP only (eGFP: n= 24; NDA-1:eGFP: n=16; p=0.0036, Fisher's exact test). Notably, all surviving NDA-1:eGFP hatchlings lost their GFP-positive cells within 2-3 weeks after hatching and thus, no stable transgenic line was obtained.

Supplementary Table 1. Statistical analysis of the minimum model for tissue and treatment as explanatory variables

Figure 3A. Abundance of *Curvibacter* sp. at various body parts
Side-by-side comparison of tissues

Body part	versus	Estimate	Std.	Error	t-value	Pr(>|t|)	p adjusted fdr
	(Intercept)	1.9979	0.1070	18.67	4.01e-14	***	5.944444e-12
	FOOT	-2.6821	0.1514	-17.72	1.07e-13	***	5.944444e-12
TENTACLE	HEAD	-1.7384	0.1514	-11.48	2.94e-10	***	2.041667e-09
	BODY DOWN	-2.0300	0.1514	-13.41	1.86e-11	***	3.100000e-10
	BODY UP	-1.9630	0.1514	-12.97	3.41e-11	***	4.059524e-10
	(Intercept)	0.2595	0.1070	2.425	0.0249	*	4.882353e-02
	BODY DOWN	-0.2916	0.1514	-1.926	0.0684		1.058824e-01
HEAD	BODY UP	-0.2246	0.1514	-1.484	0.1534		2.029101e-01
	FOOT	-0.9436	0.1514	-6.234	4.35e-06	***	1.647727e-05
	TENTACLE	1.7384	0.1514	11.485	2.94e-10	***	2.041667e-09
	(Intercept)	0.03491	0.10703	0.326	0.747666		7.675310e-01
	BODY DOWN	-0.06698	0.15136	-0.443	0.662845		7.532330e-01
BODY UP	FOOT	-0.71903	0.15136	-4.750	0.000122	***	2.346154e-04
	HEAD	0.22460	0.15136	1.484	0.153430		1.917875e-01
	TENTACLE	1.96303	0.15136	12.969	3.41e-11	***	2.131250e-10
	(Intercept)	-0.03207	0.10703	-0.300	0.767531		7.675310e-01
	BODY UP	0.06698	0.15136	0.443	0.662845		7.584039e-01
BODY DOWN	FOOT	-0.65204	0.15136	-4.308	0.000343	***	7.656250e-04
	HEAD	0.29159	0.15136	1.926	0.068383		1.058824e-01
	TENTACLE	2.03001	0.15136	13.412	1.86e-11	***	3.100000e-10
	(Intercept)	-0.6841	0.1070	-6.392	3.09e-06	***	1.647727e-05
	HEAD	0.9436	0.1514	6.234	4.35e-06	***	1.647727e-05
FOOT	BODY DOWN	0.6520	0.1514	4.308	0.000343	***	7.656250e-04
	BODY UP	0.7190	0.1514	4.750	0.000122	***	3.630952e-04
	TENTACLE	2.6821	0.1514	17.719	1.07e-13	***	5.944444e-12

Figure 3D. NDA-1 expression in hydra knockdown lines D1 and E11

General output of the statistical model

	F-statistics $F_{1,8}$	p
Treatment	93.006	1.11e-05
Line	6.757	0.03164
Line x Treatment	11.949	0.00861

Side-by-side comparison control versus knockdown

	diff	lwr	upr	p_adjusted	
D1 (kd-wt)	-0.8172679	-14,154,722	-0.2190636	0.0101530	*
E11 (kd-wt)	-17,304,441	-23,286,483	-11,322,398	0.0000696	***

Figure 3E. Body site distribution of *Curvibacter* sp. in NDA-1 knockdown lines and controls

General output of the statistical model

	χ^2 statistics ddl	Deviance	P-value
Tissue	45	9.1787	7.507e-09
Treatment	43	1.1432	0.02432

Side-by-side comparison control versus knockdown

Side-by-side	Estimate	Std.E.	t	value	p adj. fdr
Head kd-wt	0.3144	0.1354	2.312	0.025080	0.0430
Foot kd-wt	0.3144	0.1354	2.312	0.0251	0.0430

Supplementary References:

[1] Lenhoff, H. M. (1961). Activation of the Feeding Reflex in Hydra littoralis: I. Role played by reduced glutathione, and quantitative assay of the feeding reflex. *J. Gen. Physiol.* **45**, 331–344.

[2] Campbell, R. D., Josephson, R. K., Schwab, W. E. & Rushforth, N. B. *Excitability of nerve-free hydra*. *Nature* **262**, 388–390 (1976).
